# The Loco Plant

**Published:** 1888-06

**Authors:** B. F. Kingsley

**Affiliations:** 8 E. Commerce St., San Antonio, Texas


### CORRESPONDENCE.

### THE LOCO PLANT.—LETTER FROM DR. KINGSLEY.

*Editor Daniel's Texas Medical Journal:*

At different times, and in various journals, during the past year, the "Loco" plant has been discussed, showing a widespread difference of opinion, especially as regards its medical properties and therapeutic effect, which is not surprising, as little more than its proper place in botanical nosology is known.

Among other effects, the power of producing insanity is probably falaciously ascribed to it. Considerable observation of its effects on animals, shows it to act chiefly on the nervous system, producing muscular spasm and tremor, with great prostration.

Some time ago, the subject attracted the attention of Mr. James Kennedy, Ph. D., of this city, who has obtained an ample supply, and is now engaged in making a thorough study of the plant, the result of which will be published soon, with perhaps some experiments on animals, which will demonstrate its physiological and therapeutic effects.

One writer has said that it is not eaten by mules. A personal observation to the contrary, and illustrative of its toxic effects, may be not uninteresting.

While an acting-assistant surgeon in the army, en route from Fort Davis, Texas, to Fort Quitman, with some United States troops, it was noticed one morning that several of the mules were sick. While on the march that day, three of them grew much worse, and had to be taken out of harness. At first it was thought to be colic, but as the mules of the command had grazed at liberty about the spring, where "Loco" grew most, and the horses had been herded at a distance, where grass was better, the difficulty was not hard to discern. One barely dragged himself into camp, in the greatest agony, as manifested by his swolen sides, his terrible groans, and intense restlessness. He would jump, or plunge, forward, and then backward, lie down and stand up alternately, never still, until finally, with a series of forward and backward lunges, he fell broadside, dead. A rough autopsy resulted as follows:

Abdomen fearfully distended; rupture of the diaphragm, through which protruded a prodigious knuckle of intestine; guts, throughout, inflated to bursting point, glistening and bloodless, and as clean within as though flushed with water, except in the rectum, where there there was an impaction, which condition suggested a line of treatment for the others, which were almost in the same predicament. Copious injections of hot soapsuds and castor oil were resorted to, which brought away large quantities of fecal matter and gas, and a drench given of bicarbonate of soda, tincture opium and chloroform, which being repeated, subdued the pain and muscular spasm. Both recovered, though one was ill for two or three weeks.

B. F. Kingsley, M. D.,

April 21, 1888. 8 E. Commerce St., San Antonio.

---

San Antonio, Texas, May 4, 1888.

Mr. Editor—Relative to the paper on "Loco," already in your hands, and in view of the completion of the study of that plant by Kennedy, a few more amendatory items may be advantageously added. To use the language of Mr. Kennedy : "In the beginning of the investigation a cursory examination for alkaloids showed the presence of a substance reacting with solution of iodine, potassium sodo-hydrargyrate, and phospho molybdic acid. A more thorough examination, however, revealed the fact that the reactions were due to a peculiar organic acid, which, being volatile, was separated by destillation and its properties carefully studied."

Instead of its containing such active poisonous principles as heretofore ascribed to it, it is found to be quiet innocuous and free from all blame and responsibility in the numerous falacies with which its name is associated. I now believe that the terrible gaseous distention reported in the mules was nothing but an exaggerated form of flatulent colic by the rapid evolution and retention of gas. The vioient accompanying nervous symptoms are not incompatible with the colic theory, as anyone will see who has watched a horse with a sharp attack of the common disorder, and the successful treatment suggested and adopted in the cases mentioned is rather confirmatory than otherwise—of that theory. The non-toxic nature of the drug was shown by its exhibition to a dog in large quantities in the form of infusion, decoction, powder, and of the acid distillate, with no perceptible effect.

It is barely possible that a poisonous element may be developed at a later stage of the plants growth, or to perhaps a poisonous insect, but there is no evidence to this effect. Having seen its effect in horses, mules, sheep and cattle, and with a knowledge of the foregoing facts, I conclude that it is non-poisonous, and that the symtomatology and pathology arising from its use, is that of an overfeed of any unaccustomed green feed, and is that of flatulent colic.

B. F. KINGSLEY.

P. S.—Mr. K.'s paper will be read before the State Pharmaceutical Association, which meets in Austin June 12. B. F. K.

